# Epidemic Myalgia Presenting as Cervical Pain: A Case Report

**DOI:** 10.7759/cureus.55874

**Published:** 2024-03-09

**Authors:** Junki Mizumoto

**Affiliations:** 1 Department of Medical Education Studies, International Research Center for Medical Education, Graduate School of Medicine, University of Tokyo, Tokyo, JPN

**Keywords:** viral infection, trapezius muscle, sternocleidomastoid muscle, epidemic myalgia, cervical pain

## Abstract

A 53-year-old female visited our hospital because of cervical and abdominal pain preceding fever and upper respiratory symptoms. Severe tenderness was noted over the bilateral sternocleidomastoid muscles, the superior portion of the trapezius muscle, and the umbilical region of the abdomen. The patient reported exacerbation of posterior neck pain in the supine position and during the transition from sitting to the supine position. The diagnosis of epidemic myalgia was finally made. This case highlights the presence of the cervical variant of epidemic myalgia.

## Introduction

Epidemic myalgia is characterized by transient myalgia preceding fever and upper respiratory symptoms, typically occurring in individuals with a history of exposure to sick contacts [1,2]. Despite documentation of epidemic myalgia causing neck pain from the 1940s to the 1960s [3-6], this clinical presentation is frequently overlooked or disregarded in contemporary medical practice. This case underscores the manifestation of cervical pain attributed to epidemic myalgia and its distinctive physical findings.

## Case presentation

A previously healthy female at age 52 visited our hospital because of cervical and abdominal pain, fever, and cough during the early days of January in Japan. Six days before, the patient participated in a private gathering where she encountered several people exhibiting symptoms suggestive of an upper respiratory tract infection. Four days before, she developed a fever of 39.0 ℃, accompanied by cough, nasal discharge, and general malaise. The fever subsided after a brief period. One day before, she experienced intense cervical and abdominal pain. She did not take any medication.

On examination, the patient exhibited a body temperature of 37.0 ℃, with all other vital signs within normal limits. Severe tenderness was noted over the bilateral sternocleidomastoid muscles and the superior portion of the trapezius muscle. The patient reported exacerbation of posterior neck pain in the supine position with the head on a pillow and during the transition from sitting to the supine position. No signs of arthritis were observed. Additionally, tenderness was elicited over the umbilical region of the abdomen, accompanied by a positive Carnett's sign, or intensifying the pain upon the contraction of abdominal wall muscles. There were no signs of rash, hepatosplenomegaly, or discomfort in the facial region or lower extremities. Further examination revealed no abnormal findings in the pharynx, eyes, temporal arteries, or chest.

Laboratory tests revealed a leukocyte count of 9400 /µl (reference range: 3040-8540), serum C-reactive protein level of 1.54 mg/dL (reference range: 0-0.14), and erythrocyte sedimentation rate of 27 mm/h (reference range: 1-20). Other data is shown in Table 1.

**Table 1:** Laboratory data WBC, white blood cell; Hb, hemoglobin; MCV, mean corpuscular volume; PLT, platelet; AST, aspartate aminotransferase; ALT, alanine aminotransferase; LDH, lactate dehydrogenase; Cre, creatinine; BUN, blood urea nitrogen; CPK, creatine phosphokinase; CRP, C-reactive protein

Lab item	Data	Normal range
WBC	9,400/µL	3,040 - 8,540
Hb	13.2 g/dL	10.8 - 14.9
MCV	84.5	85.0 - 101.0
PLT	24.9×10^4^/µL	15.0 - 36.1×10^4^
AST	16IU/L	13 - 30
ALT	7 IU/L	7 - 23
LDH	157 IU/L	106 - 211
Cre	0.51 mg/dL	0.46 - 0.79
BUN	7.4 mg/dL	8.0 - 20.0
CPK	50 IU/L	41 - 153
Na	140 mEq/L	138 - 145
K	4.2 mEq/L	3.6 - 4.8
Cl	102 mEq/L	101 - 108
CRP	9.30 mg/dL	0 - 0.30

Urinalysis yielded no abnormal findings. A sonographic examination of the carotid arteries and contrast-enhanced CT revealed no evidence of aortitis or other pathological findings.

Based on the presence of muscle pain in the neck, shoulder, and abdomen preceding upper respiratory tract infection with exposure to sick contact, a provisional diagnosis of epidemic myalgia was established. Serologic testing conducted by commercial means failed to detect the presence of any virus. An IV administration of acetaminophen 1000 mg resulted in rapid pain relief, with the resolution of all the symptoms except cough by the following day. Four days later, the patient's condition remained stable, while her husband developed fever, cough, nasal discharge, and cervical pain. Given this clinical information, a definitive diagnosis of epidemic myalgia was confirmed.

## Discussion

According to the recent literature, two clinical subtypes of epidemic myalgia have been identified and commonly recognized. One subtype primarily manifests as pleuritic chest pain or epigastric pain and is associated with coxsackievirus B, occasionally with the involvement of echovirus and coxsackievirus A [1]. The other subtype predominantly involves the bilateral proximal upper and/or lower limbs, occasionally accompanied by orchialgia, and is linked to human parechovirus type 3 (HPeV3) [2,7]. The present case has features different from these subtypes: the main affected area was the sternocleidomastoid muscle and the superior portion of the trapezius muscle.

Probable outbreaks of epidemic myalgia presenting with cervical pain were documented in 1941 [3], 1946 [4], 1960 [5], and 1963 [6]. In the reports from 1946, 1960, and 1963, tenderness of the trapezius muscle was noted in all cases described. In the 1941 report, although details are obscure, most cases exhibited tenderness in the trapezius muscle, with occasional involvement of the deltoid or sternomastoid muscles. Subsequent reports of epidemic myalgia causing cervical pain became rare; however, a recent case of epidemic myalgia attributed to HPeV3, which affects muscles throughout the body including the sternocleidomastoid and rectus abdominis muscles, has been reported [8].

Differential diagnoses for fever and cervical pain encompass conditions such as meningitis, aortitis including carotidynia, crowned dens syndrome, and tendinitis of the longus colli muscle. However, in the present case, notable features include pronounced tenderness over the muscle in the neck and exacerbation of posterior neck pain upon assuming the supine position or transitioning from sitting to supine. The superior portion of the trapezius muscle functions to extend the head [9]. The bilateral contraction of the sternocleidomastoid muscle serves to extend the head when the cervical spine is not fixed and to flex the cervical spine and the head forward when the cervical spine is fixed [10]. These muscles are thus stressed when supine with the head on the pillow and transitioning from sitting to supine. Therefore, these distinctive characteristics may differentiate it from other potential diagnoses. Additionally, a careful examination can distinguish between pain originating from the sternocleidomastoid muscle and carotidynia: physicians should pay attention to the anatomy of the neck and check whether the painful area is limited to the sternocleidomastoid muscle or the carotid artery. Epidemic myalgia seldom exhibits specific findings on imaging tests, such as CT scans, and laboratory tests, which can lead physicians to overlook this condition. Awareness of the clinical presentation of epidemic myalgia may facilitate an accurate and cost-effective diagnosis. Most cases of epidemic myalgia show rapid improvement by non-steroidal anti-inflammatory drugs and acetaminophen [1,2,8], and the accurate diagnosis may avoid unnecessary intervention.

## Conclusions

Many contemporary physicians may acknowledge that epidemic myalgia commonly affects the chest (attributed to coxsackievirus B and other viruses) and proximal limbs (associated with HPeV3). Nonetheless, epidemic myalgia can also involve the trapezius and sternocleidomastoid muscles, leading to cervical pain. Experiencing exacerbated posterior neck pain upon assuming the supine position or transitioning from sitting to supine may suggest this cervical variant of epidemic myalgia.
